# Perioperative opioid-minimization approach as a useful protocol in the management of patients with Ehlers–Danlos syndrome-hypermobility type, craniocervical instability and severe chronic pain who are to undergo occipito-cervical fixation

**DOI:** 10.1186/s13023-023-02829-9

**Published:** 2023-07-25

**Authors:** Carlos Ramírez-Paesano, Claudia Rodiera Clarens, Allan Sharp Segovia, Alan Coila Bustinza, Josep Rodiera Olive, Albert Juanola Galceran

**Affiliations:** Servei Central d’Anestesiología (Anestalia), Centro Médico Teknon, Grupo Quironsalud, Carrer Vilana 12, 08022 Barcelona, Spain

**Keywords:** Opioid-free anesthesia, Opioid-minimization-approach, Central sensitization, Hyperalgesia, Craniocervical instability, Craniocervical fixation, Occipitocervical fixation, Ehlers–Danlos syndrome/hypermobility type, Joint hypermobility syndrome

## Abstract

Patients suffering from connective tissue disorders like Ehlers–Danlos syndrome hypermobility type/joint hypermobility syndrome (EDS-HT/JHS) may be affected by craniocervical instability (CCI). These patients experience myalgic encephalomyelitis, chronic fatigue, depression, extreme occipital-cervical pain, and severe widespread pain that is difficult to relieve with opioids. This complex and painful condition can be explained by the development of chronic neuroinflammation, opioid-induced hyperalgesia, and central sensitization. Given the challenges in treating such severe physical pain, we evaluated all the analgesic methods previously used in the perioperative setting, and updated information was presented. It covers important physiopathological aspects for the perioperative care of patients with EDS-HT/JHS and CCI undergoing occipital-cervical/thoracic fixation/fusion. Moreover, a change of paradigm from the current opioid-based management of anesthesia/analgesia in these patients to the perioperative opioid minimization strategies used by the authors was analyzed and proposed as follow-up considerations from our previous case series. These strategies are based on total-intravenous opioid-free anesthesia, multimodal analgesia, and a postoperative combination of anti-hyperalgesic coadjuvants (lidocaine, ketamine, and dexmedetomidine) with an opioid-sparing effect.

## Key points

1. Chronic neuroinflammation, neuronal damage and central sensitization are common pathways that cause severe widespread pain in patients with Ehlers–Danlos syndrome hypermobility type/joint hypermobility syndrome (EDS-HT/JHS) and craniocervical instability.
2. The use of opioid-free anesthesia with propofol, ketamine, lidocaine, and dexmedetomidine is feasible in patients with EDS-HT/JHS who are to undergo occipital/cervical fixation, and the support of a service specialized in postoperative acute pain is essential for the viability of this management.
3. Opioid-free anesthesia plus perioperative opioid-minimization management may reduce the postoperative pain in patients with EDS-HT/JHS that undergo occipital/cervical/thoracic fixation.
4. Postoperative infusions of anti-hyperalgesic coadjuvants (lidocaine, ketamine, and dexmedetomidine) along with multimodal analgesia provide a significant opioid-sparing effect. However, more studies are needed to justify their use for more than 72 h in this type of patient***.***
5. The perioperative opioid-minimization approach is a reasonable option for patients with EDS-HT/JHS suffering from gastrointestinal dysfunction, opioid-induced hyperalgesia, central sensitization and severe widespread pain.
6. In the context of the perioperative opioid-minimization approach, methadone is the most suitable opioid for use as a rescue analgesic for severe pain owing to its anti-NMDA effect and its effect of reducing the reuptake of serotonin and norepinephrine.

## Introduction

Some patients with Ehlers–Danlos syndrome-hypermobility type/joint hypermobility syndrome (EDS-HT/JHS) may suffer from Craniocervical Instability (CCI). CCI can result in continuous microtraumas and inflammation on craniocervical joint surfaces. It causes repetitive peripheral sensitization, which eventually leads to the phenomenon of central sensitization (CS) and hyperalgesia [1, 2]. These patients have complex clinical features that often consist of depression, chronic fatigue syndrome (CFS), myalgic encephalomyelitis (ME) [3], severe occipital-cervical pain, and severe widespread pain throughout the body. This pain is extremely difficult to treat and poorly controlled with opioids [4]. In addition, some of these patients with EDS-HT/JHS are opioid intolerant because they suffer from functional gastrointestinal disturbances, mast cell activation syndrome (MCAS) and autonomic symptoms like postural orthostatic tachycardia syndrome (POTS) [5].

The current perioperative anesthesia and pain management protocols followed with patients with EDS-HT/JHS and CCI who were programmed for occipital-cervical fixation/fusion (OCF) call for opioid-based approaches. Using opioid-based protocols seems to be a contradiction, considering the proposed nociceptive mechanisms and the high percentage of patients with an intolerance to opioids.

Owing to the complex clinical features of these patients and the difficulty encountered in managing their physical pain, updated information is included in this paper which covers important physiopathological considerations for the perioperative care of patients with EDS-HT/JHS and CCI that undergo OCF. Moreover, a change of paradigm from the current opioid-based management of anesthesia/analgesia in these patients to the perioperative opioid-minimization strategies used by the authors was analyzed and proposed as follow-up considerations from our previous case series. These strategies are based on total-intravenous opioid-free anesthesia (OFA-TIVA), multimodal analgesia, and a postoperative combination of anti-hyperalgesic coadjuvants (lidocaine, ketamine, and dexmedetomidine) with an opioid-sparing effect.

## Physiopathology

Due to the complex variety of symptoms, clinical signs, and additional syndromes that coexist in patients with EDS-HT/JHS and CCI who are going to undergo OCF, it is important that the perioperative team understand the proposed physiopathology that may explain the cause of all symptomatic features. Likewise, it is particularly important that anesthesiologists know that these patients frequently take a wide variety of chronic medications, some of which might provoke drug interactions with anesthetic medications and postoperative treatment for pain management (Table 1).

**Table 1:** Chronic medication frequently prescribed to patients with EDS-HT/JHS, POTS, MCAS and widespread pain. POTS and MCAS triggers

*Treatment related to POTS*
Alpha-adrenergic drugs (midodrine or dihydroergotamine)
Beta-blockers (metoprolol, atenolol, bisoprolol, or propranolol)
Clonidine (POTS-hyperadrenergic type)
Pyridostigmine
Fludrocortisone
Oral sodium/potassium supplements
Selective serotonin/norepinephrine reuptake inhibitors (SSNRI)
*Treatment related to chronic pain, anxiety, and sleep disorders*
Fentanyl (patch, oral), oral morphine, oral oxycodone, buprenorphine (patch)
Pregabaline, gabapentin,carbamazepine
Benzodiazepines. Mitarzapine,quetiapine
*Treatment related to MCAS*
Anti-H1: Cetirizine, levocetirizine, desloratadine, fexofenadine, diphenhydramine, chlorpheniramine
Anti-H2: ranitidine, famotidine
Leukotriene modifying agents (LTMA): Montelukast, zafirlukast, zileuton
Mast cell stabilizer: Cromolyn sodium, ketotifen, rupatadine, omalizumab
EpiPen (SOS)
Glucocorticoids (prednisone)
Chemotherapy: Interferon Alfa-2b, cladribine, masitinib, imatinib
*Treatment related to gastro-intestinal dysfunction*
Trimebutine, metoclopramide, domperidone,cromilyn sodium, probiotics, promethazine, omeprazole, pantoprazole, esomeprazole
Warning on management
*Medication that can worsen POTS*
Alpha adrenergic antagonists, calcium antagonist, diuretics, nitrates, hydralazine, opiates, phenothiazines, angiotensin receptor blocking agents, angiotensin converting enzyme inhibitors
*Some potential MCAS triggers*
Cold or sudden temperatures changes
Stress: emotional, environmental, physical
Pain
Drugs: NSAIDs, some opiates, some antibiotics, and contrast dyes
COX-2 inhibitors: etoricoxib or celecoxib may be safe
Chemical odors, perfumes and scents
Infections, venoms
Sun/Sunlight
Mechanical irritation, friction, vibration

COX-2 inhibitors: Cyclooxygenasa-2 selective nonsteroidal anti-inflammatory drugs

### Genetic conditions, chronic neuroinflammation and central sensitization are common pathways that cause neuronal damage and severe widespread pain [2, 4]

Patients with connective tissue diseases may suffer from craniocervical instability (CCI). These diseases include systemic lupus, rheumatoid arthritis, and genetic disorders such as Ehlers–Danlos syndrome-hypermobility type/joint hypermobility syndrome (EDS-HT/JHS), Marfan syndrome, Loeys–Dietz syndrome, Stickler syndrome, Cleidocranial dysostosis, Morquio syndrome, osteogenesis imperfecta, and Down’s syndrome. This broad group of genetic diseases characterized by generalized joint hypermobility can bring on laxity of the spinal ligaments that provoke severe symptoms due to CCI [1, 6].

Both CCI and EDS-HT/JHS can lead to the adaptation and compensation of movement patterns. Consequently, they cause overloading in other areas along with additional constant microtraumas to other joints, ligaments, and tendons of the movement apparatus, all of which are characterized by generalized soft tissue laxity. These conditions produce widespread chronic pain, which is triggered by complex noxious mechanisms like CS [7–11]. Another crucial factor related to biomechanical issues is the lack of proprioceptive acuity [12]. It has been suggested that this lack plays a significant role in causing gait abnormalities, generalized joint and soft tissue microtrauma, and musculoskeletal pain [8, 9]. Cervical medullary syndrome (CMS) may be involved in the development of more severe proprioceptive disturbances. Furthermore, CMS is considered an important medical factor that contributes to widespread severe pain in patients with CCI and EDS-HT/JHS [6].

CMS may explain some of the neurological and ancillary symptoms in patients with CCI and EDS-HT/JHS, particularly when a Chiari malformation is present. An unstable cervical spine may cause functional brainstem compression, which may be influenced by neck movements and axonal damage due to deformative stress [13]. Cervical medullary syndrome (CMS), defined as a group of bulbar symptoms and myelopathy, has been well described in a recent consensus statement on Craniocervical instability [14]. CMS may be explained by the traumatic deformation of axons that induces abnormal sodium influx through mechanically sensitive Na+ channels, which subsequently triggers an increase in intra-axonal calcium via opening of the voltage-gated calcium channel, upregulation of the glutaminergic pathway, chronic neuroinflammation, and apoptosis [4]. The most frequent clinical manifestations observed in patients with CMS are mentioned in Table 2. [6, 13]. There may also be signs of dysautonomia such as POTS, sensory loss, functional gastrointestinal disturbances, delayed gastric emptying, chronic(slow transit) constipation, rectal evacuatory dysfunction and bladder dysfunction [5, 15, 16]. Some of these symptoms coincide with those observed in chronic fatigue syndrome (CFS), myalgic encephalomyelitis (ME), or a combination of both (ME/CFS) [1, 3].

**Table 2:** The most frequent symptoms observed in patients with Cervical medullary syndrome [6, 13]

Altered vision (particularly photophobia and diplopia)
Altered hearing (peculiar misophonia)
Altered speech and swallowing
Vertigo, dizziness
Numbness (i.e., peripheral hypo/anesthesia), and
Neuropathic pain (e.g., allodynia, hyperalgesia, paresthesia, and burning sensations)
Tremulous limbs, muscle weakness
Lack of balance and coordination
Abnormal movements (e.g., fasciculations, periodic limb movements, dystonia)
Altered sleep architecture, mood changes, and emotional and cognitive disturbances (minor memory and concentration disturbances)

On the other hand, the presence of migraines, temporo-mandibular joint (TMJ) dysfunction syndrome and myofascial neck pain in patients with JHS are factors that make the diagnosis of craniocervical pain produced by CCI difficult [17, 18] which also increase pain severity. Migraines are the most common type of headache in patients with EDS-HT/JHS due to underlying arteriopathy and POTS [15, 16].

Undoubtedly, some overlap exists among all hereditary diseases of the connective tissue when considering the multi-systemic nature of generalized joint hypermobility. Interestingly, the clinical characteristics of most patients who have undergone OCF by our surgical team present with EDS-HT/JHS accompanied by CCI, chronic fatigue, severe occipital-cervical pain, severe widespread pain with a poor response to opioids, functional gastrointestinal disturbances, MCAS and autonomic symptoms like POTS [5].

On the other hand, genetic conditions in the encoding of α-tryptase presented in some patients with EDS-HT/JHS may be involved in the coexistence of symptoms that affect the skin, gastrointestinal tract, and circulatory and musculoskeletal systems [19, 20].

Studies suggest that there is a common genetic condition related to excessive germline duplications and triplications in the allelic TPSAB1 gene encoding α-tryptase that provokes an increase in serum basal tryptase levels from mast cell activity (tryptase ≥ 8.0 ng/ml). It is a common autosomal dominant inheritance that *may partially explain* the coexistence of these multi-systemic symptoms that affect the skin, gastrointestinal tract, cardiovascular and musculoskeletal systems. This might also be related to the coexistence of MCAS, POTS, gastrointestinal disorders and EDS-HT/JHS. This coexistence is not a constant phenomenon (less than 15%) and it can occur with different levels of severity [19, 20]. We think that this alteration in the allelic TPSAB1, particularly expressed by the high prevalence of intestinal dysfunction in patients with EDS-HT/JHS, might be another reason that would justify avoiding or minimizing the use of opioids in these kind of patients, beyond the concept of the multimodal analgesic approach and its benefits for nociception.

There are some opioids that are considered to have a high risk of histamine release, such as morphine, pethidine (meperidine) and codeine. These opioids should be avoided, particularly in patients with MCAS and POTS. On the other hand, there is evidence that tramadol, oxycodone, fentanyl, sufentanil and methadone have a low risk of histamine release. These opioids may be an alternative for occasional use, such as rescue analgesia [21]. In fact, some patients with EDS-HT/JHS and severe pain attend the pre-anesthetic evaluation on tramadol, fentanyl, buprenorphine, or oxycodone. Some of these patients have significant opioid-related side effects. However, due to the high risk of postoperative intestinal dysfunction, gastric dilatation, nausea, vomiting and urinary dysfunction in patients with EDS-HT/JHS, a perioperative opioid minimization approach is recommended. For this reason, we have stopped the intraoperative administration of multiple doses of fentanyl, remifentanil or sufentanil infusions.

Relative to joint laxness, the symptoms of patients overlapped in the EDS-HT and JHS groups. Experts have recently considered these two disorders as indistinguishable at the clinical level [18, 22, 23]. It is important to clarify that the terms EDS-HT and JHS are used indistinctly. Although this concept still needs a stronger genetic demonstration, we agree with recent evidence suggesting that EDS-HT and JHS might be the same disorder at the genetic level [18]. The 2017 International Classification of Ehlers–Danlos syndrome adapted from Malfait F, Francomano C, et al. is shown in Table 3.

**Table 3:** The 2017 International Classification of Ehlers–Danlos syndromes. Adapted from Malfait F, Francomano C, et al. (2017)

Clinical subtype and	IP	Gen	Protein(s)	Grupo
(Abbreviation)	Implicated	Implicated	Pathogenesis	
Classical EDS (cEDS)	AD	Major: COL5A1	Type V collagen	A
		Rare: COL1A1	Type I collagen	
Classical-like EDS (clEDS)	AR	TNXB	Tenascin XB	C
Cardiac-valvular (cvEDS)	AR	COL1A2	Type I collagen	A
Vascular EDS (vEDS)	AD	Major: COL3A1	Type III collagen	A
		Rare: COL1A1	Type I collagen	
Hypermobile EDS (hEDS)	AD	Unknown	Unknown	Unresolved
Arthrochalasia EDS (aEDS)	AD	COL1A1	Type I collagen	A
		COL1A2		
Dermatosparaxis EDS (eEDS)	AR	ADAMTS2	ADAMTS2	A
Kyphoscoliotic EDS (kEDS)	AR	PLOD1	LH1	B
		FKBP14	FKBP22	
Brittle cornea syndrome (BCS)	AR	ZNF469	ZNF469	F
		PRDM5	PRDM5	
Spondylodysplastic EDS (spEDS)	AR	B4GALT7	β4GalT7	D
		B3GALT6	β3GalT6	
		SLC39A13	ZIP13	
Musculocontractural EDS (mcEDS)	AR	CHST14	D4ST1	D
		DSE	DSE	
Myopathic EDS (mEDS)	AR or AD	COL12A1	Type XII collagen	C
Periodontal EDS (pEDS)	AD	C1R	C1r	E
		C1S	C1s	

IP, inheritance pattern; AD, autosomal dominant; AR, autosomal recessive

Groups according to pathogenetic mechanisms

Group A: defects in collagen primary structure and processing

Group B: defects in collagen folding and cross-linking

Group C: defects in structure and function of myomatrix, the interface between muscle

Group D: defects in glycosaminoglycan biosynthesis

Group E: defects in complement pathway

Group F: disorders of intracellular processes

### Are the current opioid-based protocols the wrong way to deal with these patients?

In patients with EDS-HT/JHS who develop CCI, both severe craniocervical pain and widespread pain have multifactorial causes [22]. Pain is strongly related to chronic nociceptive neuroinflammation, glial activation, and neuronal plasticity in the spinal cord, brainstem, and brain. They can be a precursor to the phenomenon of central sensitization (CS) [2, 4, 7].

Moreover, many patients with CCI, JHS, chronic fatigue, and severe chronic pain receive distinct types of opioids, which further complicate pain due to opioid-induced hyperalgesia (OIH) [2, 22, 24, 25]. From time to time, these patients may experience a category of pain known as central intractable pain. It is a painful condition that does not respond to opioids, and their use may even be detrimental to patients [4, 7, 25]. In the preoperative period, the our patients are diagnosed OIH thanks to the help of a team of neurologists and specialist in chronic pain management. It is striking that up to 50% of our patients with EDS-HT/JHS with CCI and chronic opioid treatment attend our pre-anesthetic evaluation with signs and diagnosis of OIH. Our percentage of patients with OIH seems to be very high compared to the general population under chronic opioid treatment. However, some authors have confirmed that the clinical prevalence of OIH in post-operative patients, as well as in patients with chronic non-cancer pain or chronic cancer pain, is unclear. In addition, the bulk of the references that have analyzed the prevalence of OIH are focused on experimental studies in animals. There is no consensus on the use of clinical tests to establish a diagnosis of OIH, which may lead to confusion in clinical interpretation. It is therefore difficult to make a proper comparison between our prevalence of OIH and the prevalence in the general population [26]. It could be that the prevalence presented in this article is influenced by the complex medical conditions of the patients included in our data. Furthermore, these patients are derived from other non-specialized centers suffering from advanced processes of CCI, severe chronic pain, allodynia, hyperalgesia and an inadequate pain management. Some of these patients are treated with high doses of different types of opioids without a correct diagnosis of CCI. Consequently, our team of neurologists and chronic pain specialists start a preoperative reduction of opioid doses and discontinue the simultaneous use of opioids.

As noted in other publications, it is possible that the diagnosis of withdrawal-associated hyperalgesia (WAH) was erroneously included in our data as part of the prevalence of OIH, which may be biased. Therefore, it is advisable not to extrapolate our data to the general and the entire EDS-HT/JHS population under chronic opioid medication [27]. An advanced state of physical, neurological and emotional deterioration are the clinical characteristics of our patients, many of whom were classified as merely psychiatric cases prior to our assessment.

Taking into consideration the probable mechanisms of chronic severe pain that affect some our patients with EDS-HT/JHS and CCI, and their frequent association with gastrointestinal dysfunction, the use of opioids was stopped in total intravenous anesthesia (TIVA) during OCF.

The differences were clearly visible. Therefore, we believe that the opioid-based protocol for anesthesia and pain management may be an improper way to manage EDS-HT/JHS patients with CCI and severe chronic pain who are going to have OCF.

Intraoperative opioid-based analgesia (remifentanil or sufentanil infusions) has been replaced with infusions of lidocaine, ketamine, magnesium, and dexmedetomidine. They are coadjuvants with known analgesic and antihyperalgesic properties. This anesthetic protocol is directed toward enhancing postoperative pain control, decreasing postoperative opioid rescues, and reducing preoperative opioid doses in patients who have been prescribed these medications over a prolonged period. The infusions of lidocaine, ketamine, and dexmedetomidine were continued at lower doses up to 72 h postoperatively as part of a multimodal analgesia plan (Tables 4, 5). Recently, the medical literature have supported the postoperative use of lidocaine, dexmedetomidine and ketamine infusions for up to 72 h as part of a multimodal analgesic approach for ERAS (Enhanced Recovery After Surgery) protocols for various types of surgeries [28–33]. An average reduction in length of stay (LOS) was approximately 5 days in those patients who underwent OFA plus postoperative opioid-minimization approach. The maintenance of these infusions for the mentioned postoperative periods has been feasible by having an organized acute pain management service with specialized nurses.

**Table 4:** The opioid-free anesthesia plus perioperative opioid-minimization protocol (OFA-plus) modified from *Ramirez-Paesano C, *et al

*Anesthesia*
Induction
Midazolam 0.1 mg/kg
Propofol TCI Schnider Model: Ce 3.0–4.0 mcg/ml (Bis 40–50)
Ketamine: 0.25 mg/kg
Lidocaine: 1.5 mg/kg
Cisatracurium: 0.2 mg/kg
Esmolol: 100–500 mcg/kg
Maintenance: Propofol TCI Schnider Model: Ce 2.0–4.0 mcg/ml (BIS 40–50)
Lidocaine: 2.0–3.0 mg/kg/h
Ketamine: 0.2–0.3 mg/kg/h
Dexmedetomidine: 0.2–0.3 mgc/kg/h
MgSO_4_:50 mg/kg
Dexamethasone 8 mg. Dexketoprofen 50 mg. Paracetamol 1 g
Ondansetron 8 mg
*Post-operative analgesia*
Analgesics, anti-inflammatory: Paracetamol 1 g every 8 h.Desketoprofen 50 mg e/8 h. Dexamethasone 4 mg every 12 h
Postoperative anti-hyperalgesic infusions up to 72 h
Lidocaine: 0.5 mg/kg/hr
Ketamine: 0.05 mg/kg/hr
Dexmedetomidine: 0.05 mcg/kg/hr
Other postoperative coadjuvants: Baclofen 25 mg e/hours, haloperidol 5 mg e/12 h, tizanidine 4 mg e/12 h. Ondansetron 8 mg e/8 h. Diazepam 2.5–5 mg e/8 h
Rescue for severe breakthrough pain
Methadone: 5–10 mg subcutaneous e/8 h

NMB are not allowed when EMG and MEPs are included in the neuromonitoring plan. NMB are allowed when only SSEPs is required for neuromonitoring

Haloperidol is not administered in patients with confirmed POTS

Mcg, micrograms; mg, milligrams; TCI, target-controlled infusion; Ce, effect site concentration; BIS, bispectral index, EMG, electromyography; MEPs, motor evoked potentials; SSEPs, somatosensory evoked potentials; NMB, neuromuscular blocker; MgSO_4_, magnesium sulphate

**Table 5:** Medications used in OFA plus perioperative opioid-minimization approach

Drugs	Mechanisms of actions	Clinical effect	Side effects
Lidocaine	Reduce TNF α. Decreases pro-inflmmatory cytokines Peripheral and central anti-hyperlagesic Na-channel block	Analgesia. Anti-inflammatory Anti-hyperalgesia	No reports of risk of life-threatening events Dose reduction in liver diseases To monitor in known cardiac arrhythmia and epilepsy
Paracetamol	Central inhibition of COX3 receptors Activation of serotonergic pathway Modulation of endogenous cannabinoid pathway	Analgesic	Dose reduction in liver
Magnesium	No-competitive antagonist of NMDAr Synergistic effect with Ketamine	Analgesia Anti-hyperalgesic Neuronal-glial protection Antiarrhythmic and hemodynamics Homeostatic	Prolongs the effects of NMB Contraindicated in kidney failure and heart blocks
Dexamethasone	Anti-inflammatory by nuclear mechanisms	Analgesia. Anti-inflammatory. Anti-emetic	Hyperglicemia with high dose
NSAID's (Dexketoprofen)	Peripheral and central inhibition of COX1/2 Inhibition of inflammation acting on the nucleus of glial cells	Anti-inflammatory. Analgesic Anti-hyperalgesic	Gastric toxicity (COX1) Potential kidney toxicity
Ketamine	NMDA Antagonist. Decreases pro-inflammatory cytokines by acting on KF-kB-nuclear factor-kB Anti-inflammatory cholinergic pathway	Analgesia at low doses. Anti-hyperalgesia Dose-dependent sedation	Potential psyco-mimetic effect. At low dose is well tolerated without risk of hyperexcitability, muscle hypertonia or risk of joint dislocations
Dexmedetomidine	Blocks NE release in the CNS. Acts in the Locus Ceruleus. Activate descending inhibitory pathway. Highly selective α2-agonist	Sedation. Analgesia. Anti-hyperalgesia Sympatolysis. Reduce heart rate and may decrease blood pressure*	Drowsiness but counteract hypersalivation due to Ketamine. Hypertermia^§^

TNF-α, tumoral necrosis factor alpha; COX, cyclooxygenase enzyme; NMDAr, N-methyl-D-aspartate receptor; NMB, neuromuscular blockers; NE, norepinephrine; CNS, central nervous system

*Doses of dexmedetomidine greater than 2 mcg/Kg/h may cause an increase in blood pressure

^§^It has been reported that dexmedetomidine at high doses during prolonged infusions might cause hyperthermia for reasons not well known

In certain cases of EDS-HT/JHS patients underwent OCF, these infusions were used for more than 72 h without any complication (5 days). These cases suffered from severe widespread pain with significant neuropathic symptoms, and the use of opioids was completely contraindicated for severe intestinal disorders and MCAS. These cases represented less than 2% of our case series. It is important to mention that postoperative infusion of lidocaine, ketamine and dexmedetomidine for more than 72 h is not yet supported by the literature. In our cases, the prolongation of these infusions was justified by compassionate use.

### Is there evidence for OFA plus perioperative opioid-minimization management in patients with EDS-HT/JHS undergoing OCF?

An exhaustive search for evidence related to opioid-free anesthesia and perioperative opioid-minimization management in patients with EDS-HT/JHS undergoing OCF has been carried out. Upon completion of this research, two independent reviewers (C.R-P and C.RC) screened the titles and abstracts using the Cochrane Library, Ovid Medline, PubMed Central, and Google Scholar databases according to PRISMA guidelines. Any arising differences were settled by a discussion with a third and fourth party (A.CB and A.SS). Only publications written in English since 2010 to 2022 with the following terms were applied to the search strategy: (((((hypermobile syndrome) OR (EDS-HT)) AND (Opioid-Free Anesthesia)) AND (opioid-free anaesthesia)) AND (perioperative opioid minimizing approach)) AND (multimodal analgesia)) AND (occipitocervical fixation)) AND (OCF)) OR (craniocervical fixation). Randomized clinical trials, case series, case reports, narrative review and editorials were equally included to complete the current information available on OFA plus perioperative opioid-minimization management in patients with EDS-HT/JHS undergoing OCF.

The inclusion criteria were as follows:

- Patients diagnosed with EDS-HT/JHS and CCI.
- Patients with EDS-HT/JHS and CCI undergoing OCF with opioid free-total intravenous anesthesia.
- Patients with an opioid intolerance due to gastrointestinal disorders, nauseas and vomiting, and/or OIH, and/or MCAS triggers.
- Patients under OFA-TIVA and postoperative use of lidocaine, ketamine, and dexmedetomidine infusions.

The exclusion criteria were as follows:

- Patients with CCI without EDS-HT/JHS.
- Patients with EDS-HT/JHS and CCI who underwent any other surgery than OCF or OCF plus thoracic fixation.
- Patients with EDS-HT/JHS and CCI who underwent OCF under opioid-based anesthesia/analgesia, with or without medication for multimodal analgesia.

**Fig. 1:**
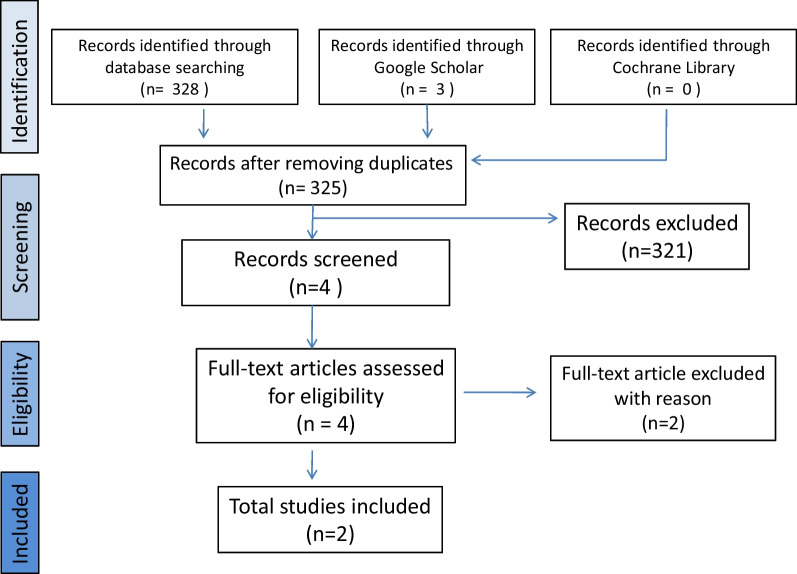
Flowchart illustrating the article selection process according to the PRISMA guidelines

The database search identified 328 records. After removing duplicate records, 324 articles were screened for relevance of titles and abstracts. Four relevant articles were reviewed for eligibility, resulting in a full-text review. Two articles were excluded. One of them is a systematic review focused solely on the surgical treatment of craneocervical instability in patients with EDS-HT/JHS. The other article deals with the use of OFA plus opioid-minimization management in various clinical settings other than OCF, including EDS-HT/JHS [34, 35]. (Fig. 1).

Two remaining articles were included in the final qualitative analysis, a case series study of 42 patients, and a narrative review [36, 37]. The articles were published in English between 2020 and 2021 and present an evidence level V.

Information related to the use of opioid-free anesthesia plus postoperative infusions of lidocaine, ketamine, and dexmedetomidine as part of opioid-minimization management in patients with EDS-HT/JHS for any type of surgery is lacking. Only one previously published study by the authors of this paper can be found, registered in ClinicalTrials.gov Identifier: NCT04437589 by Ramirez-Paesano et al. [36], entitled “Opioid-free anesthesia for patients with joint hypermobility syndrome undergoing cranio-cervical fixation: a case-series study focused on anti-hyperalgesic approach.” It matches the following keywords: (((((opioid-free anesthesia) AND (Ehlers–Danlos syndrome–hypermobility type)) AND (joint hypermobility syndrome)) AND (craniocervical fixation)) AND (multimodal analgesia)) AND (antihyperalgesia)) AND (central sensitization)). We also found a randomized study, registered in ClinicalTrials.gov Identifier: NCT03417193 by Barakat H. and Abi Raadet, entitled “Opioid-Free Anesthesia in Major Spine Surgery (posterior lumbar fusion)” in which the concept of anti-hyperalgesia is included and the intraoperative infusions of lidocaine, ketamine and dexmedetomidine are used. However, they use sevoflurane/N_2_O in their groups and the patients do not suffer from EDS-HT/JHS or any other collagen disorder.

A recently published review referred to OFA techniques followed by opioid-free analgesia such as OFAA, whose name and acronym could be synonymous with our OFA-plus protocol [38].

### Could the OFA plus postoperative opioid-minimization approach be useful in the management of these patients?

For many years, our team has managed patients with EDS-HT/JHS who underwent OCF with TIVA using opioids like fentanyl, sufentanyl, or remifentanil as intraoperative analgesics. In the past, postoperative pain management was based on morphine, hydromorphone, nonsteroidal anti-inflammatory drugs, and benzodiazepines. The postoperative period used to be a challenging time with poor outcomes in terms of pain management, gastrointestinal side effects, and patient satisfaction. Based upon a better comprehension of nociceptive physiopathology, a decision was taken to change the paradigm around the use of opioids as mandatory analgesics in the perioperative period. The new paradigm for opioid minimization management includes infusions of lidocaine, ketamine, and dexmedetomidine as coadjuvant painkillers with significant anti-hyperalgesic and opioid sparing effects. Reducing the use of opioids by managing these patients with the “OFA-plus” protocol has resulted in a reduction in Visual Analogue Scale (VAS) in terms of postoperative time (*p* < 0.001). VAS at hospital-discharge was lower in the “OFA-plus” group (*p* < 0.001).

The methadone requirement was lower in the OFA-plus group (*p* < 0.001). Among patients in the OFA-plus group, 78% (95% CI) required no methadone rescue, 8.7% required 10–15 mg/day, and 4.3% of patients required more than 15 mg/day.

In contrast, 95% (95% CI) of the OP group (group with opioid-based management) required methadone rescue at high doses. Thirty-six percent required 10–15 mg/day and 42.8% required more than 15 mg/day of methadone rescue. The OFA-plus group showed decreased ileus, nausea, and vomiting (*p* < 0.001). In the OFA-plus group, 60.9% of patients had decreased opioid requirements at hospital discharge compared with the preoperative values. However, in the OP group, 26.3% of patients maintained the same opioid requirement, 63.2% increased, and no patients showed a decrease. A 77% reduction in anxiolytic requirements was observed [36]**.**

Opioid-free total intravenous anesthesia (OFA-TIVA), an important part of the OFA-plus protocol, was provided following the protocol described in our recently published article [36]. Since then changes to our protocol has been made. During the postoperative period, only methadone is used as a rescue. Any other opioids used during the postoperative period correspond to those used chronically by the patient.

Also, the preventive use of haloperidol to reduce the incidence of ketamine-induced hallucinations have been included in patients who suffer from severe anxiety and important psychological disorders. The literature suggests that low-dose ketamine has a low potential for hallucinations. However, in our case series, the incidence of hallucinations was 17.4% despite all the patients received midazolam at 0.1 mg/kg intraoperatively. In addition, many of them were chronically medicated with benzodiazepines and antidepressants to treat severe anxiety, mood disorders, panic attacks, and depression.

This incidence of hallucinations was considered to be very high, and this fact motivated us to search for the mechanisms of ketamine-induced hallucinations. We do not have a definitive explanation for this result. We hypothesized that potential causes such as genetic, psychologic or neuropsychiatric susceptibilities may be the explaination, but it is only a speculative reasoning. In any case, we needed an optional medication to reduce the ketamine-induced hallucinations. A recent meta-analysis and systematic review suggested that the phycomimetic effects of ketamine may involve the dopaminergic system, and a mechanism of neuronal firing by dopamine release was proposed [39]. Acute administration of ketamine increases dopamine levels in the frontal cortex, striatum, and nucleus accumbens, and the unpleasant side effects of ketamine are generally thought to be mediated by its agonistic effect on dopamine DA-2 receptors [40]. On the other hand, a study in healthy humans showed that pre-treatment with oral administration of haloperidol 5 mg can reduce cognitive impairment produced by subanesthetic ketamine [41]. Moreover, there has also been evidence that haloperidol prophylaxis may be effective in the reduction of ketamine-induced emergence delirium in children [42]. Other authors have reported successful treatment of ketamine-induced agitation with haloperidol [43]. Finally, haloperidol is an antagonist of postsynaptic dopamine DA-2 receptors in the mesolimbic system, which provides additional benefits such as antiemetic, non-opioid analgesic, and sedative effects. Since the use of low-dose haloperidol has been incorporated into our practice, the incidence of ketamine-induced hallucinations has reduced to less than 5%. Haloperidol is not recommended in patients with a confirmed diagnosis of POTS. [44–48].

Table 5 shows a summary of the analgesic mechanisms of action and side effects of the medications used in our protocol.

### Should the anti-hyperalgesic infusions be continued in the postoperative period or not?

To decrease the requirement of opioids during postoperative period, we believe that it is helpful to keep the postoperative infusions of lidocaine, ketamine, and dexmedetomidine at lower doses. The goal is to attenuate the inflammatory response resulting from surgical trauma and its effect on pain, CS, and gastrointestinal function [7, 49, 50]. In our protocol, the anti-hyperalgesic infusions are maintained postoperatively for up to 72 h, monitored by anesthesiologists and the acute pain management service. When the infusions are discontinued, it may be necessary to change to oral medications with similar mechanisms of action like oral ketamine, dextromethorphan, both with anti-NMDA effect.

Many of these patients present involuntary cervical and thoracic muscle contractures and spasticity leading to exacerbation of postoperative pain. Tizanidine or baclofen are used when muscle contractures and spasticity are identified as an additional component of pain. Tizanidine is a muscle relaxant with alpha-2-agonist analgesic mechanism with anti-nociceptive effect in neuropathic pain through inhibition of pro-inflammatory cytokines production via suppression of Toll-like receptor 4/Nuclear Factor-ƙB (TLR4/NF- ƙB) activation as well as postoperative opioid-sparing effect [51, 52].

Baclofen, a gamma-aminobutyric acid type B receptor agonist, blocks the release of excitatory neurotransmitter in neurons and glial cells (glutamate and aspartate) by interfering with voltage-gate calcium channels. Baclofen decreases muscle tone and prevents reflex muscle contraction and spasticity [53].

An improvement in those patients with “uncontrolled pain” by using oral baclofen (25 mg e/8 h) has been noticed. This fact led us to consider cervical-dorsal muscular spasticity or muscle contracture as an additional component of pain crisis. [54, 55].

Methadone, an opioid with anti-NMDA effect, is used as a painkiller rescue to treat postoperative breakthrough pain [56]. So far, there has been no MCAS complication following methadone rescue despite its potential to release histamine [36].

Later, the administration of oral ketamine may be continued in patients with widespread pain that is difficult to control. This is also the case if there is a preoperative history of treatment with opioids, OIH, an important neuropathic component, or a propensity to receive high opioid rescue doses.

## Discussion

### Are we really on the road to managing EDS-HT/JHS patients with CCI who are to undergo OCF?

Many articles on the advantages of OFA have been published. However, studies of OFA in patients that have undergone spinal surgery have revealed controversial findings relative to postoperative outcomes [57–59]. The majority of those authors only agree on the benefit of the perioperative use of non-opioid coadjuvants in the context of multimodal analgesia to achieve an enhanced recovery after spinal surgery [60–62].

A balanced combination of lidocaine, dexmedetomidine, ketamine and MgSO4 has antinociceptive and anti-hyperalgesic effects. However, there are still doubts about the analgesic efficacy of its use in OFA when compared to opioids. Doubts have also arisen as to the most appropriate combination of these coadjuvants to replace opioids. The absence of dependable nociceptive monitors has been an obstacle to changing the paradigm in spine surgery [63]. Monitoring of nociception based on the measurement of the sympathetic/parasympathetic balance in response to surgical stress is not adequate for OFA. Monitoring of nociception by measuring heart rate variability and vagal tone (HRV) may be useful to evaluate intraoperative opioid-induced analgesia (i.e., Analgesia Nociception Index (ANI), Nociception Level Index (NoL-Index)), but not for OFA**.** On the contrary, the nociceptive flexion reflex threshold (NFRT) may be more fitting to monitor nociception in patients under opioid-free total intravenous anesthesia (OFA-TIVA) without neuromuscular blockers (NMB). NFRT, a method based on electromyography (EMG) and bispectral index (BIS), is able to predict movement as a response to surgical pain under propofol monoanesthesia. When using OFA-TIVA, NFRT might be useful in predicting movement due to surgical pain [64].

This article deals with the topic of a propofol-based OFA in a group of specific patients (EDS-HT/JHS) with widespread pain and SC that have undergone major spinal surgery to treat CCI. Intraoperative analgesia is delivered by means of a balanced combination of lidocaine, ketamine, MgSO4 and dexmedetomidine. These "opioid substitutes" have anti-hyperalgesic and anti-inflammatory properties as well as multiple mechanisms of analgesic action. In addition, anesthesia is provided without neuromuscular blockers (NMB) because our surgical team requires continuous evaluation with neurophysiologic monitoring that includes motor evoked potentials (MEPs) and electromyography (EMG). Somatosensory evoked potentials (SSEPs) are also monitored. In our practice, EMG recordings have confirmed the absence of movements related to the surgical stimulus in any patient. The dosage of lidocaine, ketamine, MgSO4 and dexmedetomidine used in our OFA-plus protocol (Table 4) provides an adequate level of antinociceptive synergism, which coincides with recent publications that describe these coadjuvants as the best alternative to replace or minimize the use of opioids [65–67].

Many factors have led to disagreements about OFA for complex spinal interventions in terms of reducing postoperative opioid requirements and better recovery. Studies with very liberal inclusion criteria that include a broad range of spine surgeries, various levels of complexity of cases, and the exceptionally varied usage of coadjuvants for postoperative multimodal analgesia have made it difficult to reach a consensus on the advantages of OFA in major spinal surgery. However, there is convincing evidence that opioid‐inclusive anesthesia does not reduce postoperative pain but is associated with more side effects than OFA, particularly in patients who have a risk of gastrointestinal and bladder dysfunction, MCAS, POTS, opioid intolerance and OIH [36, 59, 68].

Ever since the “OFA-plus” protocol has been in use, improved pain control has been seen in patients. In addition, the postoperative visual analog scale (VAS) has been significantly reduced and methadone rescue has been reduced. Moreover, the postoperative comfort of the patients, gastrointestinal side effects and the need for anxiolytics drugs decreased [36].

Therefore, we believe that we are on the right pathway to providing better management of these patients*.*

Some resistance to the LA effect has been noted in regional anesthesia techniques for dental, orthopedic, and obstetric procedures. However, there is no evidence that peripheral nerve blocks or spinal/epidural anesthesia are ineffective. On the contrary, regional anesthesia may be the better choice for some orthopedic and obstetric procedures in patients with EDS-HT/JHS. On the other hand, there are some case reports of resistance to certain LA with local infiltration [69–71]. Resistance to LA appears to occur due to changes in LA dispersal because of the peculiar characteristics of EDS-collagen fibers. What’s more, certain alterations in the voltage-gated sodium channels (VGSC) in pain signaling can causes some change in the LA binding sites, resulting in a reduction of its local effect [72–74].

At the moment, our OFA-plus protocol does not currently include regional techniques with local anesthetic for postoperative analgesia. However, we believe that in the future the use of some techniques such as ultrasound-guided interfascial blocks would be possible and beneficial to further reduce methadone rescue. Further study will be necessary to test this hypothesis.

So far, any sign of resistance or the absence of an analgesic effect from intravenous lidocaine has not been observed. This may be because the mechanisms of intravenous lidocaine analgesia are mediated by a strong systemic anti-inflammatory effect and multiple antinociceptive pathways other than Na+ channel blockade-mediated analgesia at therapeutic plasma concentrations [49, 65, 66, 74].

The coexistence of widespread chronic pain, CS, opioid intolerance, OIH, gastrointestinal disturbances, and the potential presence of MCAS has been the reason for the use of OFA-plus in these patients [4, 5, 24, 25]. Movement related to the surgical stimulus is a subcortical reflex phenomenon. The use of neuromonitoring, the maintenance of hemodynamic stability and the absence of movements during the surgery have allowed us to confirm that the doses of non-opioid coadjuvants proposed in our anesthetic protocol provide adequate clinical analgesia. On the other hand, it is well known that surgical trauma triggers a local and systemic inflammatory response that intensifies in the first postoperative days. The severity of the postoperative pain may depend on this inflammatory state, which is proportional to the extent of the surgery and its duration. Considering the above, we decided to continue the postoperative use of lidocaine, ketamine, and dexmedetomidine infusions with a progressive lowering of the doses up to 72 h to overcome the peak inflammatory surgical response and its impact on pain and CS [7, 50].

If necessary, we use methadone to relieve severe breakthrough pain in the postoperative period. However, including the use of non-opioid options that affect different nociceptive pathways (medicinal cannabinoid rescues, baclofen, tizanidine, memantine, haloperidol or dextromethorphan) remains important to reduce the use of postoperative opioids [75–78]. Because methadone is still used as a rescue pain reliever, our management should not be considered an opioid-free anesthesia/analgesia protocol (OFAA) but rather an OFA plus postoperative opioid-minimization approach. Our protocol will be a completely perioperative opioid-free protocol when a new and more effective non-opioid rescue analgesic comes out and replaces methadone [79].

Moreover, at present, no analgesic can completely replace opioids during the postoperative period as rescue to control severe breakthrough pain.

We are in complete agreement with some authors that OFA should not be taken as a popular (“fashionable”) trend to be followed in any type of surgery under general anesthesia [79]. We firmly believe that multimodal analgesia plus the postoperative use of anti-hyperalgesic infusions are primarily focused on an opioid-minimizing approach. However, we also believe that the "OFA-plus" protocol may be helpful for patients with EDS-HT/JHS who are suffering from severe pain that will undergo OCF or other major surgical procedures that have a significant painful component.

### Is it suitable to use some opioids as postoperative rescue in OFA and keep denominating it an opioid-free technique?

Regarding pain management in patient with OIH and CS, we believe that methadone is the most suitable rescue opioid to treat severe pain owing to its anti-MNDA effect. Methadone decreases OIH and attenuates central sensitization. It also reduces the reuptake of serotonin and norepinephrine. Furthermore, the use of methadone with ketamine (both anti-NMDA) shows a synergy that enhances the opioid-sparing effect [80].

Patients that have undergone major surgical procedures frequently require postoperative opioids to relieve breakthrough pain control. The OFA-plus protocol used in our hospital includes methadone only as a rescue agent for severe postoperative pain that is difficult to control with non-opioid analgesics [36].

Recent literature has recommended the use of methadone (0.15–0.2 mg/kg bolus) at the beginning of anesthetic induction in complex spinal surgery [81]. Methadone offers a strong postoperative opioid-sparing effect and enhances pain control. These advantages seem to last for months following surgery compared to other opioids like morphine or hydromorphone [82].

A recent meta-analysis confirmed the benefits of methadone use at the onset of anesthesia in extensive and painful surgeries [83, 84].

If methadone is needed as a postoperative rescue, nausea and vomiting may be a problem. At some point, fosaprepitant may be an option for the management of intractable nausea and vomiting. Currently, our protocol does not include the use of methadone in the intraoperative period, which is consistent with the OFA concept. If a single dose of methadone is administered at the beginning of our OFA protocol, the anesthetic technique should be called modified OFA-plus, or simply not called OFA.

## Conclusions

Patients with EDS-HT/JHS and CCI experience severe chronic widespread pain and hyperalgesia, which are strongly related to the central sensitization phenomenon. The use of the “OFA-plus” protocol is feasible and safe to use in patients who are to undergo OCF with EDS-HT/JHS associated with severe pain, POTS, MCAS, and gastrointestinal dysfunction.

The infusions of lidocaine, ketamine, and dexmedetomidine in combination with propofol for TIVA without NMB seem to provide adequate analgesia. The neuromonitoring (BIS, EMG, MEPs and SSEPs) records suggest that this management is appropriate.

Postoperative pain management in patients who are to undergo OCF is difficult and complex. The use of lidocaine, ketamine, and dexmedetomidine infusions during the postoperative period may be helpful in improving pain management while reducing the total amount of opioids used during the hospital stay.

To date, it has been difficult to eliminate the use of opioid rescues to control severe postoperative breakthrough pain in its entirety. It is important to continue studying non-opioid therapeutic options to reduce gastrointestinal side effects, OIH, and opioid tolerance and dependence.

Finally, the last questions we should answer are: (1) *Does OFA offer short-term benefits in the postoperative period?*: In response to this it should be said that there are no randomized studies to give a definitive answer. However, at present we could say that OFA plus perioperative opioid-minimization approach reduce the total amount of opioid and the gastrointestinal side-effects in this patients. (2) *Does “OFA-plus” offer long-term benefits regarding the pain?:* To this question it is important to state that there are no studies that support it. (3) In these types of patients undergoing OCF, *Could the use of a single dose of methadone be recommended at the beginning of the anesthetic induction?* This could be possible. However, there are no studies using “the modified-OFA” in this context.

## Data Availability

Datasets used and/or analyzed during the current study are available from the corresponding author on reasonable request.

## Abbreviations

ANI: Analgesia Nociception Index
BIS: Bispectral Index
Ce: Concentration in the effect compartment
CCF: Craniocervical fixation/fusion surgery
CCI: Craniocervical instability
CCU: Critical care unit
CFS: Chronic fatigue syndrome
CMS: Cervical-medullary syndrome
CS: Central sensitization phenomenon
EDS-HT/JHS: Ehlers–Danlos syndrome hypermobility type/joint hypermobility syndrome
ERAS: Enhanced recovery after surgical protocols
HR: Heart rate
GABA: Gamma-aminobutyric acid
K+ A: Potassium channel type
LA: Local anesthesia
JHS: Joint hypermobility syndrome
MCAS: Mast cell activation syndrome
ME: MyalgicEncephalomyelitis
MEPs: Motor-evoke potentials
NFRT: Nociceptive flexion reflex threshold
NMDAr: N-methyl-d-aspartate receptors
NMB: Neuromuscular blocker
NK-1r: Neurokinin-1 receptors
NSAIDs: Nonsteroidal anti-inflammatory drugs
NoL-Index: Nociception Level Index
PCA: Patient-controlled analgesia
OCF: Occipitocervical fixation/fusion surgery
OFA: Opioid-free anesthesia
OFAA-plus: Opioid-free anesthesia and analgesia plus postoperative infusions of lidocaine, ketamine, and dexmedetomidine
OFA-TIVA: Opioid-free total intravenous anesthesia
OIH: Opioid-induced hyperalgesia
PKC: Protein kinase C
PONV: Postoperative nausea and vomiting
OP: Opioid-based anesthesia and analgesia management
POTS: Postural orthostatic tachycardia syndrome
SD: Standard deviation
SSEPs: Somato-sensitive-evoked potentials
TCI: Target-controlled infusion
TMJ: Temporomandibular joint
VAS: Visual analog scale score
VGSC: Voltage-gated sodium channel
WAH: Withdrawal-associated hyperalgesia
